# Downregulation of miRNA-590 and miRNA-182 in Early-Stage Mycosis Fungoides in Skin of Color Patients

**DOI:** 10.7759/cureus.90926

**Published:** 2025-08-25

**Authors:** Rania H Mahmoud, Mohamed El-Khalawany, Rania Abdelghani, Sameh K Fawzi, Shymaa E Ayoub, Noha E Mohamed

**Affiliations:** 1 Medical Biochemistry and Molecular Biology, Faculty of Medicine, Fayoum University, Faiyum, EGY; 2 Dermatology and Venereology, Faculty of Medicine for Boys, Al-Azhar University, Cairo, EGY; 3 Dermatology and Venereology, Faculty of Medicine for Girls, Al-Azhar University, Cairo, EGY; 4 Dermatology, Sexually Transmitted Diseases (STDs) and Andrology, Faculty of Medicine, Fayoum University, Faiyum, EGY; 5 Dermatology, Venereology, and Andrology, Armed Forces College of Medicine, Cairo, EGY

**Keywords:** early stage, lymphoma, mirna-182, mirna-590, mycosis fungoides

## Abstract

Background: Mycosis fungoides (MF), especially in skin of color and early stages, lacks specific and sensitive biomarkers for advanced understanding of its pathogenesis, diagnosis, and prognosis. This study aimed to investigate whether miRNA-590 and miRNA-182 have a role in the pathogenesis of early-stage MF and their potential diagnostic and prognostic values in skin of color patients.

Methods: This single-center prospective cohort study with a two-year follow-up included 30 adult Egyptian patients with early MF (IA-IIA) at the time of diagnosis and 20 healthy controls. Serum levels of both miRNA-590 and miRNA-182 were detected in patients at the time of diagnosis relative to controls. Cutaneous lymphoma international prognostic index and clinical progression risk were also determined. Follow-up was conducted every six months over a two-year period to detect disease progression.

Results: There was a statistically significant downregulation of both miRNA-590 and miRNA-182 in patients at the time of diagnosis compared to controls (p = 0.01). Additionally, there were statistically insignificant correlations between serum levels of both miRNA-590 and miRNA-182 and the two-year outcome (p = 0.4 and 0.5, respectively).

Conclusion: This study highlighted the potential dual role (oncogenes/tumor suppressors) of both miRNA-590 and miRNA-182 in the development of early MF and the lack of their progression prediction values in skin of color patients. However, further larger-scale studies are required.

## Introduction

Mycosis fungoides (MF) is an extranodal non-Hodgkin lymphoma, and it is the most prevalent variant of primary cutaneous T-cell lymphomas (CTCLs), accounting for approximately 75% of cases, with an annual incidence rate of one per 100,000 person in the United States [1,2]. MF has a higher prevalence and greater impact on quality of life in the skin of color population (phototypes ≥IV) compared to the White population [3,4]. MF is characterized by the presence of skin lesions, including patches, plaques, or tumors containing epidermotropic malignant CD4+ CD45 RO+ helper/memory T-cells [5]. In its early stages, MF is slowly progressive with a favorable prognosis; however, advanced disease that constitutes one third of cases could be disseminated with lymph nodes, blood, and visceral involvement, a condition that is associated with approximately 40-45% five-year survival [6,7]. MF in skin of color patients has diverse clinicopathological presentations and heterogeneous outcomes [8]. Black women with early-onset MF have more progressive disease and a higher mortality rate compared to White women [9].

Several studies reported various molecular changes in MF, including chromosomal, genomic, and gene expression aberrations; however, no clear disease-specific changes have been reported [10]. Furthermore, there is a lack of studies investigating genetic, immunological, or molecular differences that could underlie racial disparities in MF [11]. Therefore, there is a lack of specific and sensitive biomarkers that enhance understanding of pathogenesis, diagnosis, and prognosis of MF, especially its early stages [12], which remain challenging to predict high-risk patients [13], especially those with skin of color.

MicroRNAs (miRNAs) are small (18-22 nucleotides) non-coding, single-stranded RNA molecules with several biological activities, cellular and extracellular. Expression of miRNAs changes in different disease states, including MF. These expression changes result in transcriptional regulation and posttranscriptional changes together with cellular signaling [14]. Several studies explored that dysregulated microRNAs (miRNAs) have a signature in pathogenesis, diagnosis, progression of MF, and patients' survival. Oncogenic miRNAs, e.g., miRNA-106b, are upregulated in MF while tumor suppressor miRNAs, e.g., miRNA-195-5p, are downregulated. Other miRNAs, such as miRNA-122 and miRNA-214, are prognostic markers. Additionally, serum levels of miRNA-155, miRNA-203, and miRNA-205 could be used as diagnostic markers with an accuracy of more than 90% [15-17].

miRNA-590 is reported to have an important role in cellular proliferation, differentiation, and the occurrence of tumors [18]. Expression of miRNA-590 is found to be significantly upregulated in renal cell carcinoma [19], cervical cancer [20], hepatocellular carcinoma [21], breast cancer [22], and cancer of the vulva [23], indicating an oncogenic role for miRNA-590 [24]. miRNA-182 is reported to have a dual role (both oncogenic and tumor suppressor) in several malignancies, e.g., renal cell carcinoma, bronchogenic carcinoma, and melanoma. This role is achieved through its effects on various aspects, including apoptosis, metastasis, proliferation, and drug resistance [24].

In spite of data reporting the role of both miRNA-590 and miRNA-182 in several tumors, there are no available data exploring their role in MF in skin of color patients. Furthermore, it was reported that dermatology publications relevant to skin of color, diversity, and inclusion are low [25].

Accordingly, we conducted this prospective cohort study with a two-year follow-up to investigate whether miRNA-590 and miRNA-182 have a role in the pathogenesis of early-stage MF and their potential association with disease outcome for a better understanding of the molecular pathogenesis of MF in skin of color patients and possibility to be future therapeutic targets.

## Materials and methods

Participants

This single-center prospective cohort study was carried out in the Dermatology and Venereology Department, Kobry El-Qubba Medical Complex, Cairo, Egypt, during the period from July, 2021 to August, 2023.

We recruited 30 adult Egyptian patients with early MF and skin phototype IV or higher as study cohorts and 20 healthy volunteers who were age- and sex-matched to serve as controls.

Inclusion criteria of study cohorts

We included adult patients (≥18 years old), both sexes who had early-stage MF that was defined as stages IA-IIA (<IIB) confirmed by clinical, immuno-, and histopathological findings. The patients were diagnosed and staged according to the International Society for Cutaneous Lymphomas/European Organization of Research and Treatment of Cancer proposal [26].

Exclusion criteria

We excluded patients with advanced MF stage (≥IIB) [26] at the time of diagnosis, pediatric cases and pregnant and lactating women, patients with systemic illness associated with mi-RNA changes as renal and hepatic carcinomas, patients with other types of lymphomas and patients with any dermatologic diseases other than MF, e.g., chronic inflammatory diseases as psoriasis.

Methods

Study cohorts who were diagnosed and staged as having early MF were subjected to the collection of the following data:

1. The cutaneous lymphoma international prognostic index (CLIPi) was calculated for each patient by giving a point for each adverse prognostic factor identified for early-stage (clinical predictors of early MF progression). These factors include age >60 years, male sex, presence of plaques (stage IB), folliculotropic disease, and lymph node stage N1/N0 [27].

2. Risk scores of early MF progression (clinical progression risk) were then calculated by summing the number of adverse prognostic factors present in each patient and categorizing them according to the total score as low risk (0-1), intermediate risk (2), and high risk (3-5) [27].

3. Treatment at the time of diagnosis, including skin-directed therapy (e.g., phototherapy) and/or systemic therapy (e.g., methotrexate or acitretin).

4. Estimation of serum levels of both miRNA-590 and miRNA-182 at the time of diagnosis for both study cohorts and controls.

5. Two-year outcome data were collected to detect whether MF remained in its early stage, improved, or progressed according to clinical, immuno-, and histopathological findings. Improvement was defined as either complete recovery or regression of the MF stage, e.g., from stage IIA to stage IA. Progression was defined as progression from early MF stages to advanced stages (≥stage IIB) and/or death due to this progression.

6. Estimation of serum levels of both miRNA-590 and miRNA-182 for both study cohorts and controls.

Samples collection

Six-milliliter blood samples were withdrawn from both patients and controls, which were collected into two tubes: one tube held ethylenediaminetetraacetic acid (EDTA) for the complete blood count (CBC), while the other was allowed to clot for fifteen minutes before being centrifuged at four thousand g for ten minutes to extract the serum. The serum was then stored at -80°C for all serological tests, including those involving molecular biology techniques.

RNA extraction

RNAs were isolated from serum using Qiagen (Valencia, CA, USA) extraction kits in accordance with the manufacturer's instructions. Nano Drop2000 (Thermo Scientific, Waltham, USA) was used to measure the RNA concentration and purity.

Reverse transcription reactions

The total RNA was subjected to reverse transcription in a final volume of 20 μL. Using the RevertAid First Strand cDNA Synthesis Kit (Thermo Fisher Scientific, USA), RNAs were reverse transcribed by reverse transcription-polymerase chain reaction (RT-PCR) kit into cDNAs in accordance with the manufacturer's instructions.

Quantitative real-time polymerase chain reaction

Using the miScript SYBR Green PCR Kit (Qiagen, Germany), quantitative RT-PCR was used to amplify up the cDNA templates. Using the specified miRNA-590 primer (catalogue number YP00205448 Lot. Number 201705190377-1) and miRNA-182 primer (catalogue number YP00206070 Lot. Number 201803060144-2), the total volume for each reaction involving miRNA-590 and miRNA-182 was 25 μL. For the purpose of relative quantification and normalizing the expression of miRNA-590 and miRNA-182, SNORD 68 was employed. For both, the qRT-PCR was set up with the following cycling parameters: First, the reaction was activated for 15 minutes at 95°C. Next, the Rotor-gene qRT-PCR system thermocycler (Qiagen, USA) was used to cycle through denaturation for 15 seconds at 94°C, annealing for 30 seconds at 55°C, and extension for 30 seconds at 70°C. We have repeated these steps for 40 times. The relative expression of RNAs was determined using the 2-ΔΔCt technique.

Statistical analysis

Data were statistically described in terms of mean ± standard deviation (± SD), median and range, or frequencies (number of cases) and percentages when appropriate. Numerical data were tested for the normal assumption using the Kolmogorov-Smirnov test. Comparison of numerical variables between the study groups was done using the Mann-Whitney U test for independent samples. For comparing categorical data, the Chi-square (χ²) test was performed. An exact test was used instead when the expected frequency is less than 5. Correlation between various variables was done using the Spearman rank correlation coefficient. Two-sided p-values less than 0.05 were considered statistically significant. IBM SPSS version 22 (Statistical Package for the Social Sciences; IBM Corp, Armonk, NY, USA) for Microsoft Windows was used for all statistical analyses.

## Results

**Figure 1:**
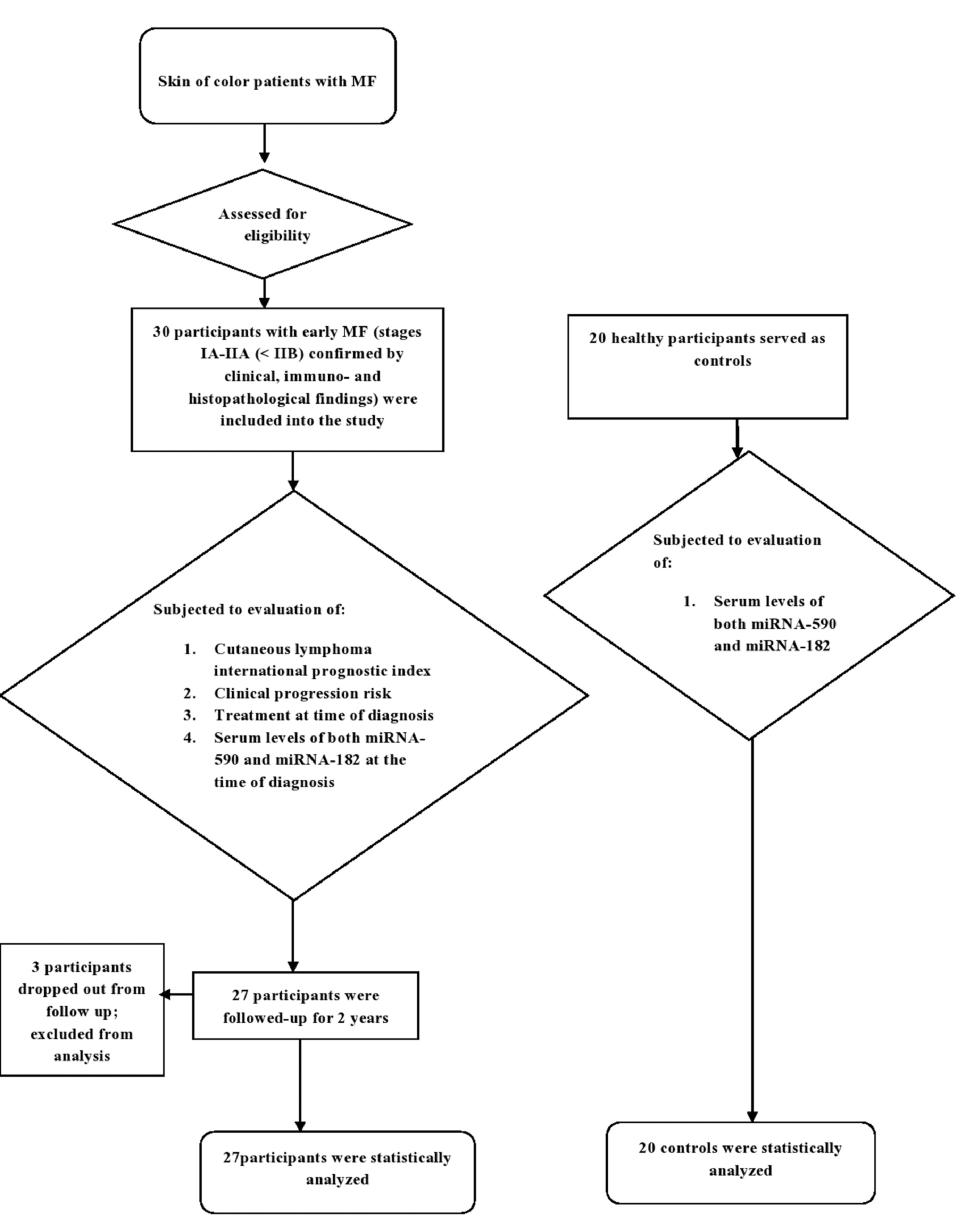
Study flow diagram. MF: mycosis fungoides.

This prospective exploratory cohort study included 30 adult Egyptian patients with early MF; three patients dropped out during follow-up, and only 27 patients were statistically analyzed (Figure 1). Their skin phototypes ranged from IV to V.

Demographic data

Among study cohorts, 17 patients (63%) were men and 10 patients (37%) were women. Their ages ranged from 21 to 58 years old, with a mean of 36 years ± 12.5 SD. Healthy controls were 14 men (70%) and six women (30%); their ages ranged from 22 to 60 years, with a mean of 39.2 years ± 12.2 SD. There were statistically insignificant differences between patients and controls regarding both sex and age (p-value = 0.51, Pearson Chi-square test = 0.4, Mann-Whitney U test, respectively).

Therapeutic strategies

At the time of diagnosis, 23 patients (85.2%) had stage IB and received phototherapy (narrowband-ultraviolet B (NB-UVB) or Psoralen plus ultraviolet-A (PUVA)), three patients (11.1%) had stage IIA and received systemic therapy (methotrexate or acitretin) combined with PUVA phototherapy, and one patient (3.7%) had stage IA and received PUVA phototherapy.

Progression index and clinical progression risk

Regarding CLIPi and clinical progression risk, 17 patients (63%) had CLIPi = two with intermediate progression risk, nine patients (33.3%) had CLIPi = one with low progression risk, and one patient (3.7%) had CLIPi = zero with low progression risk. The clinical progression risk of all patients ranged from low to intermediate.

Serum levels of both miRNA-590 and miRNA-182 and their correlation to the outcome, progression index, and clinical progression risk. Regarding miRNA-590, the patients demonstrated a mean serum level of 2.7 µL ± 5.3 SD, ranging from 0.04 to 24.7 µL, while the mean serum level of miRNA-182 in patients was 2.1 µL ± 2.9 SD, ranging from 0.13 to 10.2 µL. There was statistically significant downregulation of both miRNA-590 and miRNA-182 in patients with a mean rank of 19.93 µL at the time of diagnosis relative to controls with a mean rank of 29.5 µL (p-value = 0.01, Mann-Whitney U test).

Regarding the two-year outcome, 26 patients survived at the end of two years, and 24 patients (88.9%) were improved. The improved patients had disease stages ranging from IA to IIA, their CLIPi ranged from 0 to 2, and their clinical progression risk ranged from low to intermediate risk. Three patients (11.1%) progressed. The progressed patients had CLIPi = 2 with intermediate progression risk; of them, two patients (66.6%) were on NB-UVB phototherapy and progressed from stage IB to stage IIA, and one patient (33.3%) was on methotrexate and PUVA phototherapy for stage IIA and died because of progression to tumor stage. There were statistically insignificant correlations between serum levels of both miRNA-590 and miRNA-182 and the two-year outcome (p-value = 0.4 and 0.5, respectively, Spearman's rho test). Furthermore, there were statistically insignificant correlations between serum levels of both miRNA-590 and miRNA-182 and different early MF stages, CLIPi, and clinical progression risk, as demonstrated in Table 1.

**Table 1:** Correlations between serum levels of both miRNA-590 and miRNA-182 and different early MF stages, CLIPi, progression risk, and two-year outcome. CLIPi: cutaneous lymphoma international prognostic index, MF: mycosis fungoides. Spearman's rho test. p-value < 0.05 was considered statistically significant.

Variable	miRNA-590 serum level		miRNA-182 serum level	
	Correlation coefficient/Spearman's rho test	p-value	Correlation coefficient/Spearman's rho test	p-value
MF stage (n=27)	-0.08	0.69	-0.04	0.82
CLIPi (n=27)	-0.12	0.53	-0.2	0.29
Progression risk (n=27)	-0.09	0.64	-0.19	0.32
Two-year outcome (n=27)	-0.15	0.45	-0.1	0.59

## Discussion

There are several studies exploring the roles of different miRNAs with different expressions in the pathogenesis, diagnosis, and prediction of progression of MF. These dysregulated miRNAs include miRNA-106b, miRNA-195-5p, miRNA-122, miRNA-214, miRNA-155, miRNA-203, and miRNA-205 [18-24]; however, the research field of the molecular background of MF is still expanding, especially in skin of color patients.

We conducted this single-center prospective cohort study with a two-year follow-up to identify whether miRNA-590 and miRNA-182 have a role in the pathogenesis of early-stage MF and their potential association with disease outcome for a better understanding of the molecular pathogenesis of MF in skin of color patients and the possibility to be future therapeutic targets.

According to this study, both serum levels of miRNA-590 and miRNA-182 were significantly downregulated in patients at the time of diagnosis relative to healthy controls (p-value = 0.01).

This finding could indicate the potential roles of both dysregulated miRNA-590 and miRNA-182 in the development of early MF. Significant downregulation of miRNA-590 in early MF in the current study is contradicted by studies exploring its significant upregulation and oncogenic role in renal cell carcinoma, cervical cancer, hepatocellular carcinoma, breast cancer, and cancer of the vulva [18-22]. This oncogenic role is suggested to be achieved by miRNA-590-induced cellular proliferation and inhibition of both cellular differentiation and apoptosis contributing to tumor development [18].

This contradiction could be explained by the possibility of miRNA-590 being a tumor suppressor whose downregulation contributes to disease development based on its role in inhibition of angiogenesis, tumor growth, and metastasis, as supported by Zhou et al. [23] in colorectal carcinoma.

Another explanation is that miRNA-590 is an oncogene and its downregulation maintained the disease in its early stage and lowered its progression over a period of two years by reduction of tumorigenesis. This rationale raised the suggestion of a dual role of miRNA-590 (oncogene and tumor suppressor) in MF and highlighted the need to further research to validate this suggestion.

Similarly, significant downregulation of miRNA-182 in early MF in this study could be explained by the possibility of miRNA-182 to be tumor suppressor that its downregulation contribute to disease development based on its role in inhibition of migration, invasion, and survival of malignant cells by regulating different signaling pathways as supported by Yu et al. in gastric carcinoma [28]. miRNA-182 could be oncogenic based on its role in enhancing proliferation and invasion of malignant cells, together with its association with poor survival, as supported by Zhao et al. in breast cancer [29]. Accordingly, downregulation of miRNA-182 explored in this study could maintain the disease in its early stage and lower its progression over a period of two years.

This dual role of miRNA-182 in early MF in the current study is supported by accumulating studies illustrating its dual role in melanoma [30] and other tumors, e.g., colorectal carcinoma, breast cancer, and gastric carcinoma [24].

According to this study, there were insignificant correlations between serum levels of both miRNA-590 and miRNA-182 and different early MF stages, CLIPi, clinical progression risk, and two-year outcome, indicating a lack of progression prediction values of both miRNA-590 and miRNA-182. This finding requires further studies with a large sample size and long follow-up periods to frame out the actual progression predictive values of both miRNA-590 and miRNA-182.

Significant downregulation of both miRNA-590 and miRNA-182 in early MF highlighted in this study could be added to other significantly downregulated tumor suppressor miRNAs confirmed by previous studies, e.g., miRNA-195-5p [15].

This study highlighted the potential role of both miRNA-590 and miRNA-182 in the pathogenesis of early-stage MF in skin of color patients. Our findings could enhance the data concerning genetic mechanisms beyond the pathogenesis of MF in skin of color patients.

This study had some limitations, including a relatively small sample size, a short follow-up period, and being single centered, which could limit the generalizability of the results. Additionally, the cited references concerning miRNAs had no focus on skin of color patients. Further studies from multiple centers with large sample size and long follow-up period are needed to validate the dual role of both miRNA-590 and miRNA-182 in early MF and the accuracy of their serum levels as potential diagnostic biomarkers and/or progression predictors in early MF in skin of color patients. Moreover, future studies are needed to compare the expressions of both miRNA-590 and miRNA-182 in early and advanced MF and possibility of being therapeutic targets in skin of color groups.

## Conclusions

This study provided preliminary exploratory results concerning the potential dual role (oncogenes/tumor suppressors) of both miRNA-590 and miRNA-182 in the development of early MF and the lack of their association to disease outcome in skin of color patients. These findings could enhance the data concerning genetic mechanisms beyond the pathogenesis of MF in skin of color patients. Further large studies are still needed to detect the accuracy of both miRNA-590 and miRNA-182 as potential diagnostic biomarkers and/or progression predictors in early and advanced MF among skin of color patients and pave the way towards emerging of innovative therapy that improve patients' survival.
